# Gene Targeting of Mouse *Tardbp* Negatively Affects *Masp2* Expression

**DOI:** 10.1371/journal.pone.0095373

**Published:** 2014-04-16

**Authors:** Samar Dib, Shangxi Xiao, Denise Miletic, Janice Robertson

**Affiliations:** Tanz Centre for Research in Neurodegenerative Diseases, University of Toronto, Toronto, ON, Canada; International Centre for Genetic Engineering and Biotechnology, Italy

## Abstract

Amyotrophic Lateral Sclerosis (ALS) is a devastating adult onset neurodegenerative disease affecting both upper and lower motor neurons. TDP-43, encoded by the *TARDBP* gene, was identified as a component of motor neuron cytoplasmic inclusions in both familial and sporadic ALS and has become a pathological signature of the disease. TDP-43 is a nuclear protein involved in RNA metabolism, however in ALS, TDP-43 is mislocalized to the cytoplasm of affected motor neurons, suggesting that disease might be caused by TDP-43 loss of function. To investigate this hypothesis, we attempted to generate a mouse conditional knockout of the *Tardbp* gene using the classical Cre-loxP technology. Even though heterozygote mice for the targeted allele were successfully generated, we were unable to obtain homozygotes. Here we show that although the targeting vector was specifically designed to not overlap with *Tardbp* adjacent genes, the homologous recombination event affected the expression of a downstream gene, *Masp2*. This may explain the inability to obtain homozygote mice with targeted *Tardbp*.

## Introduction

Amyotrophic Lateral Sclerosis (ALS) is an adult-onset and invariably fatal neurodegenerative disease characterized by the loss of motor neurons in the brain and spinal cord. The most common neuropathological feature of ALS is the presence of ubiquitinated inclusions (UBIs) in the cytoplasm of affected motor neurons [1], [2]. The nature of the inclusions remained largely unknown until a remarkable discovery identified the TAR DNA binding protein (TDP-43) as a major component in UBIs of ALS and also of Frontotemporal Lobar Degeneration (FTLD-TDP) [3], [4]. TDP-43 positive inclusions are present in both sporadic and familial forms of ALS and FTLD [3], [4] and have also been identified in other neurodegenerative diseases, such as, Parkinson [5], Alzheimer [6], Huntington [7] and inclusion body myopathies [8]. Although mutations in the gene encoding TDP-43, *TARDBP*, are relatively infrequent (<1% of total ALS cases), TDP-43 pathology is common to >90% of ALS cases, including those caused by mutations in the recently identified gene, *C9orf72* [9].

TDP-43 is ubiquitously expressed and is normally localized to the nucleus where it exerts multiple functions in transcription and pre-mRNA splicing [10]. However, the cytoplasmic location of pathological TDP-43 suggests that disease might be caused by a loss of nuclear function. To this end, *Tardbp* knockout mice have been generated to investigate this loss-of-function hypothesis, however homozygote mice were embryonic lethal, dying between 3.5 to 8.5 days of embryonic development, while heterozygotes did not display any motor neuron pathology [11], [12]. To circumvent this problem we attempted to generate *Tardbp* conditional knockout mice using the classical Cre/loxP system, flanking exons 2 and 3 with loxP sites. Although we successfully generated viable and fertile heterozygote mice with the targeted allele, we were unable to obtain homozygotes. This was not due to effects of the targeting event on reducing TDP-43 expression, thereby mimicking a knockout mouse, but instead we show that the targeting event affected the expression of a downstream gene, *Masp2*. This effect on *Masp2* could underlie the inability to obtain homozygous mice with targeted *Tardbp*.

## Materials and Methods

### Generation of *Tardbp* targeting vector

**Figure 1:**
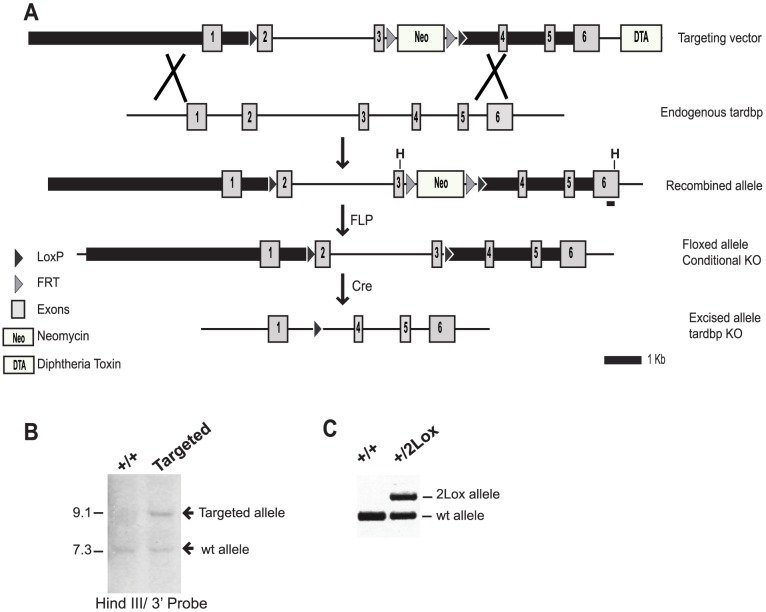
Strategy and validation of the conditional deletion of *Tardbp* gene. A) To delete exons 2 and 3 in the endogenous *Tardbp* we used a targeting vector with 6 Kb and 4 Kb homology arms, shown as black bars. The neomycin resistance was used for positive selection in embryonic stem cells and was flanked by two FRT sites to allow its removal upon FLP mediated recombination. A DTA cassette allowed negative selection of ES cells bearing random integration of the targeting vector. Upon homologous recombination in ES cells (Xs) the endogenous gene was replaced with the targeted cassette. FLP mediated recombination generated a conditional knockout where exons 2 and 3 were flanked by loxP sites. B) Southern Blot analysis of control (+/+) and targeted ES clones. A HindIII digest produced fragments of 7.3 Kb for the WT and 9.1 Kb for the targeted allele. C) Confirmation of neomycin cassette excision by PCR amplification.

To generate a conditional knockout of the *Tardbp* gene a mouse bacterial artificial chromosome (BAC) clone containing *Tardbp* (RP23-29102) was obtained from the Children's Hospital Oakland Research Institute (CHORI, https://bacpac.chori.org/) and modified by recombineering. First, RP23-29102 was made proficient for recombination by electroporation of PSC101gbaA plasmid encoding the recombination machinery. Following integration of a Zeo cassette in intron 1 of the *Tardbp* gene, a loxP site was exchanged with the Zeo cassette and placed as the most 5′ loxP recombination site, upstream of exon 2. A neomycin resistance cassette flanked by FLP recognition target (FRT) sites was amplified by Polymerase Chain Reaction (PCR) and integrated downstream of *Tardbp* exon 3. Finally, the modified gene was transferred to a plasmid containing the diphtheria toxin cassette to generate the targeting construct (Figure 1).

### Screening of ES clones and mouse genotyping

The *Tardbp* targeting vector was electroporated into C57BL6/129 embryonic stem (ES) cells at the Toronto Centre for Phenogenomics (http://www.phenogenomics.ca/). Initial identification of positive ES cell clones was performed by ethanol precipitation of genomic DNA (gDNA) and PCR amplification using primers specific for the most 5′ loxP site. We found 8 positive ES clones which were subsequently expanded in 24 well plates and screened for recombination of the 5′ and 3′ homology arms by sequencing and Southern blot analyses, respectively. The sequence of the 5′ and 3′ FRT sites flanking the neomycin cassette on all 8 ES clones was verified. For Southern blots, 15 µg of gDNA was digested overnight with HindIII, run overnight on 0.8% agarose gels and transferred by capillarity to a Hybond-N+ nylon membrane (GE Healthcare). Prehybridization for 2 hours with ULTRAhyb Ultrasensitive Hybridization Buffer (Ambion) was followed by hybridization overnight using a radioactively labeled probe for detection of the endogenous *Tardbp* gene.

### Mouse Breeding

All protocols were conducted in accordance with the Canadian Council on Animal Care and approved by the University of Toronto Faculty of Medicine and Pharmacy Local Animal Care Committee as well as the University of Toronto Animal Care Committee. ES clones with the correct recombination event were used to obtain germline-transmitting chimeras by morula aggregation at the Toronto Centre for Phenogenomics (http://www.phenogenomics.ca/). Deletion of the neomycin selection cassette was achieved by breeding the F1 *Tardbp* targeted mice with Actin-FLP mice (Jackson laboratory, #003800). Excision of the neomycin cassette was verified using PCR flanking primers.

### Quantitative Real Time PCR

Mice at 5 months of age were euthanized in a CO_2_ chamber and tissues were dissected and immediately snap-frozen in liquid nitrogen. Total RNA extraction was performed using RNAeasy kit (Qiagen); 2 µg of total RNA was used to synthesize cDNAs with oligodT and Superscript III Reverse Transcriptase (Life Technologies). Real Time PCR was performed in 20 µl reactions using SYBR Green ER qPCR SuperMix Universal (Life Technologies) according to manufacturer's instructions for the ABI7500 instrument (Applied Biosystems). cDNA was diluted 1/10 and 1/20 and all the reactions were performed in triplicates. *Tardbp* transcripts were measured using primers specific for exons 5 (forward) and 6 (reverse) respectively. TDP_RT_F1: 5′GTGGTAGATGTCTTCATTCCCAAACC; TDP_RT_R1: GATCAAATCCTCTCCACAAAGAGACTG. *Masp2* transcripts were measured with primers that discriminate between *MAp19* and *Masp2* splicing variants. The sequence of the primers is as follows: Masp_ex3_F: CTCCACCAGAACAAGCACAC; Masp_ex3_3UTR_R: CTGACTGGTATCTGACTCACTGG for *MAp19* splicing variant; and Masp_ex6_F: GTGGAGACGCACCCTGAAGCC; Masp_ex7_R: AGCGTCTTCCCACAAAATGGGCC for *Masp2* splicing variant. *Actb* gene, encoding for β-Actin, was used as a reference gene to normalize *Tardbp*, *MAp19* and *Masp2* transcript quantification, β-Actin_RT_F: GAACCCTAAGGCCAACCGT;β-Actin_RT_R: GAGGCATACAGGGACAGCACAG. Statistical differences between quantitative real time PCR samples were assessed by GraphPad software (www.graphpad.com).

### Immunoblots

Mouse tissues (brain, spinal cord, liver) were dissected, snap-frozen and homogenized in SDS/Urea Buffer (0.5% SDS/8M Urea in Phosphate Buffered Saline) for TDP-43 immunodetection or RIPA Buffer (50 mM Tris-HCl pH 7.5, 150 mM NaCl, 1% NP-40, 0.25% Na deoxycholate, 1 mM EDTA, 0.1% SDS, Protease Inhibitor Cocktail P8340 from Sigma diluted 1∶50) for Masp-2 immunodetection. Homogenates were analyzed on 10% (w/v) SDS-polyacrylamide gels and transferred to PVDF membranes which were then blocked for 1 hour at room temperature with 5% (w/v) skim milk powder in Tris-buffered saline (TBS). The membranes were then incubated overnight at 4°C with primary antibodies diluted in blocking solution as follows: mouse anti-mannan-binding Lectin (MBL) monoclonal antibody (MAB 3808) at 1∶5000 dilution; anti-TDP-43 antibody (10782-2-AP, Proteintech) diluted 1∶2000; or anti-Actin clone C4 (MAB 1501) diluted 1∶5000. Immunoblots were then washed with TBS containing 0.05% Tween 20 and incubated for 1 hour at room temperature with anti-mouse or anti-rabbit IgG conjugated to peroxidase diluted 1∶5000 in blocking solution. Antibody binding was detected with Western Lightning Plus ECL detection system (Perkin Elmer).

## Results

To generate a conditional knockout of *Tardbp* we used the classical loxP-Cre recombination system. Using BAC recombineering technology, two loxP sites were introduced flanking exons 2 and 3 (Figure 1A). Deletion of exons 2 and 3 prevents translation from both the first translation start site in exon 2 and an alternate translation start site located in exon 3.

Homologous recombination between the targeting vector and the *Tardbp* locus in ES cells generated the *Tardbp* targeted allele. The targeting event was verified in ES clones surviving neomycin selection by Southern blot and sequencing (Figure 1B). ES clone 3G was empirically selected to generate germ-line transmitting chimeras which were crossed to C57BL/6 mice. In order to remove the flipped neomycin selection cassette, F1 *Tardbp* targeted mice were crossed to Actin-FLP mice and neomycin excision was verified by PCR amplification using primers flanking the FRT sites (Figure 1C). Heterozygote mice bearing floxed exons 2 and 3 at the *Tardbp* locus were designated as *Tardbp* ^+/2lox^.

Genotyping of 71 live-born F2 mice from a cross between *Tardbp* ^+/2lox^ heterozygotes showed 22 *Tardbp* ^+/+^, 49 *Tardbp* ^+/2lox^, and no *Tardbp* ^2lox/2lox^. The absence of *Tardbp* ^2lox/2lox^ offspring was highly significant (p<0.0001, Chi-square test); the ratio of *Tardbp* ^+/+^ to *Tardbp* ^+/2lox^ mice (22∶49) fit the expected 1∶2 ratio, consistent with a purely recessive inheritance lethality in the homozygote. PCR genotyping of embryos at E12.5 and E8.5 days revealed no homozygotes, suggesting that lethality occurred prior to these time points, but was not further investigated.

**Figure 2:**
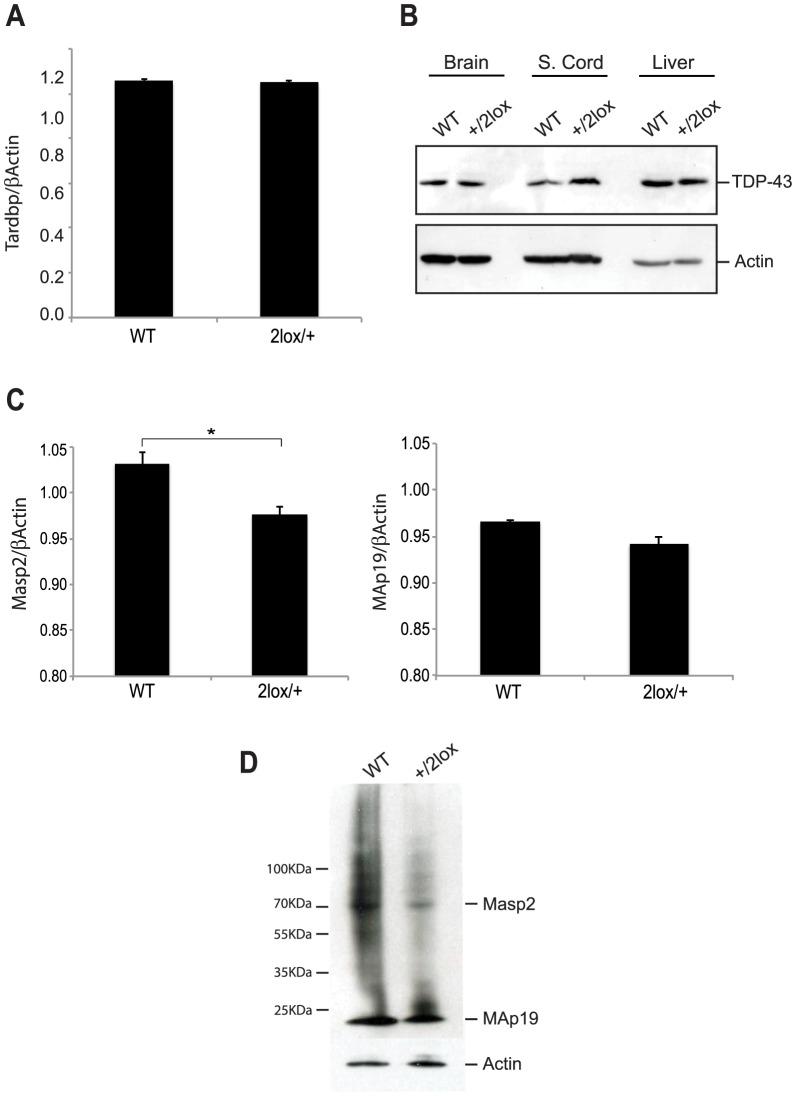
Analysis of *Tardbp* and Masp2 expression in Tardbp^2Lox/+^mice. A) Quantification of mRNA in mouse lumbar spinal cord by Real-Time PCR. No significant difference was found between *Tardbp* transcripts between WT and Tardbp^+/2lox^ mice (n = 4). B) Immunoblot analysis of TDP-43 protein expression in mouse brain, spinal cord, and liver shows no reduction in expression in *Tardbp* ^+/2lox^ mice compared to wildtype littermates. Actin was used to normalize the amount of protein loaded. C) Quantification of *Masp*2 splicing variants mRNA in mouse liver by quantitative Real-Time PCR. *Masp2* long variant was significantly reduced in *Tardbp* ^+/2lox^ compared to WT (p = 0.03, n = 3). No significant difference was found for *MAp19* transcript levels between WT and Tardbp^+/2lox^ mice. D) Analysis of total protein extracts from mouse liver by Western blot. Masp2 is clearly reduced in Tardbp^+/2lox^ mice livers when compared to WT, while MAp19 expression is marginally reduced.

Since it has previously been shown that homozygous *Tardbp* knockout mice are embryonic lethal [11]–[13], it is possible that the inability to obtain homozygote *Tardbp* ^2lox/2lox^ mice was due to an inadvertent effect of the gene targeting event on the expression of *Tardbp*. Analysis of *Tardbp* mRNA transcripts in lumbar spinal cord revealed no diminishment in TDP-43 expression between *Tardbp* ^+/2lox^ mice and wildtype littermates and this was verified at the protein level by immunoblot analysis of brain, spinal cord and liver tissue (Figure 2A, 2B). However, since TDP-43 can auto-regulate its own expression levels the possibility that the targeting event disrupted *Tardbp* expression cannot be excluded [14], [15].

An alternate explanation for the absence of *Tardbp* ^2lox/2lox^ homozygote mice could be an indirect effect occurring as a consequence of the homologous recombination event at the *Tardbp* locus. Even though the homology arms of the targeting vector were specifically designed not to overlap with adjacent gene sequences, it is possible that the homologous recombination event affected the expression of neighboring genes.

In the mouse genome, the 3′ untranslated region (UTR) of the *Tardbp* gene overlaps with the 2 last exons of the *Masp2* gene. The *Masp2* gene encodes for two isoforms. MAp19 is the short isoform (19 KDa), with the last exon located 14.5 Kb downstream of *Tardbp* 3′UTR. A longer isoform (74 KDa) called Masp2, has the last two exons overlapping 3.5 Kb with *Tardbp* 3′UTR.

To investigate this hypothesis we used qRT-PCR to analyze the expression levels of *Masp2* and *MAp19* transcripts in the liver, where it is highly expressed, of *Tardbp* ^+/2lox^ and WT littermates (Figure 2C). A significant reduction in expression of the *Masp2* transcript was observed in *Tardbp* ^+/2lox^ mice compared to WT littermates, and this correlated with a ∼50% reduction in Masp2 protein levels, as revealed by immunoblot analyses of replicate tissue samples (Figures 2C and 2D). A marginal but not significant reduction in *MAp19* transcripts was also observed (Figure 2C), that correlated with a slight reduction in MAp19 protein levels (Figure 2D). These results show that targeting of the *Tardbp* locus affected expression from the adjacent gene, *Masp2*.

## Discussion

In this investigation we generated heterozygous mice for a conditional knockout of the *Tardbp* gene by flanking exons 2 and 3 with loxP recombination sites. We found that while heterozygote *Tardbp^+/2lox^* mice were viable and fertile, we were unable to obtain homozygotes. Since *Tardbp* knockout mice are embryonic lethal [11]–[13], we checked whether there was a loss of TDP-43 expression from the targeted allele. There was no apparent reduction in TDP-43 expression at either the mRNA or protein level, which might be real or a result of auto-regulation of the *Tardbp* wildtype allele [14], [15], as has been reported in other *Tardbp* heterozygote knockout mice [11]–[13]. We also considered whether the targeting event disrupted adjacent genes. *In silico* design of the targeting vector is one of the most important steps in generating gene knockouts in mice. Since homologous recombination of a targeting vector is a rare event [16], our targeting vector was designed with long homology arms to increase the frequency of targeted integration at the *Tardbp* locus [17], while avoiding overlap with genes located upstream and downstream of *Tardbp*. The 5′ homology arm of the targeting vector (Chr4: 148.625.922–148.631.862) was 5.9 Kb long and the 3′ homology arm (Chr4: 148.617.735–148.621.728) 3.9 Kb; the latter is located 2.23 Kb downstream of the 3′UTR of *Masp2*, and as such would not be expected to affect expression of this gene. Nevertheless, we found a significant reduction in both mRNA and protein expression of the long isoform of Masp2 in heterozygote *Tardbp^+/2lox^* mice compared to their non-transgenic littermates, indicating that the targeting did affect *Masp2* and that this could underlie the inability to obtain homozygous Tardbp^2lox^ mice. Of note, the greater effect on Masp2 compared to MAp19 might relate to the overlap of the last 2 exons of *Masp2* with the 3′UTR of *Tardbp*.

The reduced expression of Masp2 could be the result of an introduction of unexpected mutations in the chromosome by the recombination event at the *Tardbp* locus. Even though homologous recombination generally occurs with high fidelity [18], many investigations have reported the incorporation of mutations upon targeted homologous recombination [19]–[21]. Moreover, the fidelity of homologous recombination can be compromised when the sequences of the targeting vector and the chromosome are not isogenic [18]. In our study, the RP23-29102 BAC used to make the targeting vector had a C57BL/6J background while the mouse Embryonic Stem cells were from a hybrid 129/C57BL6 background. It is possible that the homologous recombination event at *Tardbp* introduced mutations that altered regulatory sequences governing the expression of the *Masp2* gene. Even though regulatory sequences have been identified upstream of the *Masp2* gene [22], [23], the regulation of this gene remains poorly understood. Some of the *Masp2* regulatory enhancers could reside downstream of the gene, as has been reported for other genes [24], [25].

*Masp2* deficiency in mice confers protection from gastrointestinal ischemia/reperfusion injury and are viable in a homozygous state [26], which would seem contradictory to our inability to obtain homozygote *Tardbp^2lox/2lox^ Masp*2 deficient mice. However, this discrepancy could be due to the mouse strain background of the previously reported Masp2 deficient mouse, which was established in 129/Sv embryonic stem cells, whereas *Tardbp^+/2lox^* mice were in a hybrid C57BL6/129 strain background. It is well recognized that the strain background has a relevant influence in phenotype of transgenic mice [27], [28].

There are relatively few publications on the generation and use of *Tardbp* conditional knockout mice for the study of ALS [29]–[31]. Chiang et al., 2010 floxed *Tardbp* exon 3 and found that ubiquitous deletion of *Tardbp* leads to a metabolic phenotype in embryonic stem cells. Wu et al., 2012 showed that deletion of *Tardbp* exons 2 and 3 in heterozygote mice leads to an age dependent progressive motor dysfunction. However, to date neither group has reported on homozygote mice. Iguchi et al., 2013 obtained homozygote mice with a conditional deletion of *Tardbp* exon 2, after backcrossing for at least 5 generations, and showed an age dependent motor dysfunction. Thus, generation of TDP-43 deficient mice has been a challenging task and in this investigation we show for the first time an effect of targeting *Tardbp* on downstream gene *Masp2*.
